# Inflexibility in Reasoning: Comparisons of Cognitive Flexibility, Explanatory Flexibility, and Belief Flexibility Between Schizophrenia and Major Depressive Disorder

**DOI:** 10.3389/fpsyt.2020.609569

**Published:** 2021-01-18

**Authors:** Chen Zhu, Nate Tsz-kit Kwok, Tracey Chi-wan Chan, Gloria Hoi-kei Chan, Suzanne Ho-wai So

**Affiliations:** ^1^Department of Psychology, The Chinese University of Hong Kong, Hong Kong, China; ^2^Prince of Wales Hospital, Hong Kong, China; ^3^Alice Ho Miu Ling Nethersole Hospital, Hong Kong, China

**Keywords:** flexibility, cognitive bias, transdiagnostic, reasoning, appraisal

## Abstract

**Introduction:** Inflexibility in reasoning has been suggested to contribute to psychiatric disorders, such as explanatory flexibility in depression and belief flexibility in schizophrenia. However, studies tended to examine only one of the flexibility constructs, which could be related to each other, within a single group of patients. As enhancing flexibility in thinking has become one of the psychological treatment goals across disorders, this study aimed to examine three constructs of flexibility (cognitive flexibility, explanatory flexibility, and belief flexibility) in two psychiatric groups.

**Methods:** We compared three groups of participants: (i) 56 outpatients with a schizophrenia-spectrum disorder and active delusions, (ii) 57 outpatients with major depressive disorder and at least a moderate level of depression, and (iii) 30 healthy controls. Participants were assessed on symptom severity and flexibility, using the Trail-Making Task, the Attributional Style Questionnaire, the Maudsley Assessment of Delusions Scale (MADS) and the Bias Against Disconfirmatory Evidence (BADE) Task.

**Results:** Cognitive flexibility was reduced in the two clinical groups compared to controls. Explanatory flexibility was comparable across groups. The three groups differed in belief flexibility measured by MADS but not by the BADE task. Response to hypothetical contradiction was reduced in the delusion group than the other two groups, and the ability to generate alternative explanations was reduced in the delusion group than healthy controls.

**Discussion:** We found an effect of diagnosis on cognitive flexibility, which might be confounded by differences in intellectual functioning. Reduced belief flexibility tended to be specific to delusions.

## Introduction

Cognitive approaches stress how biassed reasoning and appraisals may explain psychopathology [e.g., (1–5)]. Some researchers focus on maladaptive belief contents about one's external and internal experiences, such as attribution biases (6) and meta-worry (7, 8). In comparison, others focus more on how individuals formulate or maintain their thoughts. Such dysfunctional processes tend to include dichotomous thinking (9, 10), inadequate information gathering (11, 12), difficulty in evidence integration (13), etc. Flexibility in reasoning is one cognitive process that has received research attention in recent years, which in its broadest sense refers to the cognitive capacity to adaptively respond to changing contexts (14, 15). A failure to sensitively respond to different circumstantial factors in thinking, reasoning, and reflecting has been reported in various psychiatric disorders.

Cognitive flexibility refers to the ability to selectively focus on accessible mental sets in response to varied task requirements (15–17). As typically measured by the Wisconsin Card Sorting Task (WCST) (18), the Trail-Making Task (TMT) (19) and other set-shifting tasks [e.g., intra-inter dimensional task; (20, 21)], cognitive flexibility manifests in the faster and more accurate grasp of the task rule when it changes, and reduced time cost in shifting between sequences.

Recent reviews suggested a deficiency in cognitive flexibility across diagnostic categories (22–24), including schizophrenia (25, 26), major depressive disorder (14), autistic spectrum disorders (27, 28), and obsessive-compulsive disorder (29). Studies that examined cognitive flexibility between diagnostic groups had yielded mixed results. For example, Mahurin et al. (30) reported significantly more errors and longer completion time in patients with schizophrenia than patients with depression on the TMT. In contrast, Moritz et al. (31) found no difference between their schizophrenia and depression samples on neither TMT nor WCST, both performing worse than non-clinical controls. As cognitive flexibility may be directly influenced by several cognitive capacities including working memory, inhibitory control, and digit span (30, 32, 33), a more precise differentiation on how cognitive flexibility tends to be compromised in light of various diagnoses may enrich process-based research of psychopathology.

As a narrower concept than cognitive flexibility, explanatory flexibility refers to the responsiveness to contextual features when forming causal attributions (34–36). The underlying premise is that individuals are able (and expected) to take into account specific situational factors, hence leading to different ways of explaining the causes of different situations. Explanatory flexibility is typically measured by the standard deviation of stability and globality of attributions for negative events on the Attributional Style Questionnaire (35, 37), although some other studies had also included internality of attributions (36, 38).

Most research on explanatory flexibility centres around depression. It has been shown that reduced explanatory flexibility interacted with adverse life events to predict subsequent depressive symptoms (35). A higher level of explanatory flexibility, on the contrary, was associated with better adjustment and less relapse in patients (35, 38, 39). Explanatory flexibility tended to decrease in response to negative mood induction, especially in individuals with a history of major depressive disorder (MDD) (34). A handful of studies compared explanatory flexibility between psychiatric disorders, questioning the specificity of compromised explanatory flexibility in MDD. Fresco et al. (40) found reduced explanatory flexibility among college students with a self-reported generalised anxiety disorder than controls. Lackner et al. (41) found more reduced explanatory flexibility in patients with MDD, generalised anxiety disorder, and adjustment disorder than other psychiatric groups. In the only study that investigated explanatory flexibility among individuals with psychosis, Silverman and Peterson (38) reported a comparable level of explanatory flexibility in patients with schizophrenia as in patients with MDD. This result was yet to be replicated.

Belief flexibility refers to the metacognitive capacity of reflecting on one's own beliefs, changing them in light of reflection and evidence, and generating and considering alternatives (42, 43). Belief flexibility has been commonly assessed through a clinical interview, where an individual's idiosyncratic beliefs are discussed with an interviewer. There are three signs of belief flexibility: (1) when one acknowledges the possibility of being mistaken, (2) when one lessens their conviction in face of hypothetical and contradictory evidence, and (3) when one can generate new and alternative beliefs about their experience (44, 45). So et al. (46) found that these three measures of belief flexibility load on the same factor. Other researchers measured belief flexibility using the Bias Against Disconfirmatory Evidence (BADE) task (47, 48), where respondents are presented with standardised and hypothetical scenarios and are asked to rate the plausibility of various explanations when new evidence unfolds. Belief flexibility is operationalised on the BADE task by the change between initial and later plausibility ratings. Other self-report measures have also been recently used to capture the construct of belief flexibility, including Davos Assessment of Cognitive Biases Scale (49), Beck Cognitive Insight Scale (50), and Fast and Slow Thinking Questionnaire (51). As self-report relies largely on self-awareness and retention, doubts about the sensitivity of measuring belief flexibility using self-report questionnaires have been raised (51).

Research on belief flexibility has taken root in psychosis, where belief flexibility has been shown to be negatively associated with severity and conviction of delusions (42, 52) and may co-vary with delusions over time (46, 53). Sanford et al. (54) and Speechley et al. (48) compared BADE performance between individuals with high-delusional schizophrenia, individuals with low-delusional schizophrenia, individuals with another psychiatric disorder (obsessive-compulsive disorder and bipolar disorder, respectively), and healthy controls. Both studies found that BADE performance distinguishes the high delusional sample from the other groups. To our best knowledge, there have been no studies comparing belief flexibility between patients with schizophrenia and patients with unipolar depression, although Everart et al. (55) found in a community sample that those with a higher score of depression or social anxiety tended to be less flexible on the BADE task. In view of the specificity of belief flexibility to psychotic delusions, it has been argued that belief flexibility may be a putative mechanism of change in intervention for delusions (42, 52, 56).

In summary, at least three constructs of flexibility have been studied in psychiatric populations. Cognitive flexibility concerns the accuracy and speed of responding to changing task demands, explanatory flexibility concerns taking into account contextual factors when attributing causes to negative events, and belief flexibility concerns reviewing one's own beliefs. While evidence has accumulated for explanatory flexibility in depression and belief flexibility in psychotic delusions, it is not clear whether the same flexibility constructs are relevant across disorders. Development of the RDoC framework has led to an increase in emphasis on identifying similarities and differences in etiological factors underlying various disorders, which may inform the transdiagnostic application of process-based intervention (57, 58). Besides, while these flexibility constructs are measured using different tools, they represent the extent to which one's reasoning shifts when confronted with new information, and it remains unclear how these flexibility constructs may be related to one another. For example, Eifler et al. (59) and Riccaboni et al. (60) found that belief flexibility as measured by BADE was positively associated with cognitive flexibility, but Moritz et al. (61) found no association between the two. There has been no investigation of explanatory flexibility with either cognitive or belief flexibility. Exploring the inter-relationship between flexibility constructs will help to deepen our understanding of the cognitive structure of inflexibility in appraisal.

This study aimed to examine three constructs of flexibility together across two psychiatric groups. The two chosen groups were outpatients with MDD and schizophrenia-spectrum disorder with delusions because these two groups have been shown to have inflexible thinking. Key hypotheses were as follows:

1. Compared to healthy controls, there will be reduced cognitive flexibility in both the Delusion group and the Depression group
2. Compared to healthy controls, there will be reduced explanatory flexibility in both the Delusion group and the Depression group.
3. Compared to healthy controls and the Depression group, there will be reduced belief flexibility in the Delusion group

We also explored the associations between the flexibility constructs across groups.

## Materials and Methods

The clinical groups were drawn from a randomised-controlled trial on the effect of metacognitive training on cognitive bias in schizophrenia and MDD (62). Data included in this study were collected at baseline (i.e., before training). The clinical trial was registered with the https://clinicaltrials.gov/ Protocol Registration and Results System (NCT03449394) by the US National Library of Medicine (NLM). Ethics approval was obtained from the Joint Chinese University of Hong Kong - New Territories East Cluster Clinical Research Ethics Committee (2014.031) and the New Territories West Cluster Research Ethics Committee (NTWC/CREC/18040).

### Participants

The sample consisted of two clinical groups and a healthy control group. All participants were aged between 18 and 65. Inclusion criteria for the Delusion group were (i) a diagnosis of schizophrenia spectrum disorder, and (ii) presence of active delusions at the time of assessment [scoring ≥ 3 on item P1 of the Positive and Negative Syndrome Scale (PANSS)] (63). Inclusion criteria for the Depression group were (i) a diagnosis of MDD, and (ii) at least a moderate level of depression [total score ≥ 20 on the Beck Depression Inventory-II (BDI-II)] (64). Exclusion criteria for both clinical groups were as follows: drug-induced or organic psychosis, bipolar disorder, a primary diagnosis of substance misuse, learning disability (FSIQ < 70), previous participation in cognitive/reasoning training program, psychotic depression, and depression with psychotic features. The control group consisted of age- and education-matched individuals who did not have any psychiatric diagnosis. Patients were recruited from hospitals via referral from the clinical team. Healthy controls were recruited from the community in Hong Kong through advertisements at educational institutions, churches, public transport stations, and vocational training centres.

### Measures

Psychiatric diagnoses were ascertained by the Chinese-bilingual Structured Clinical Interview for DSM-IV Axis I Disorders (65). Level of depression in the Depression group was confirmed by the BDI-II (64).

#### Clinical Symptoms

For the Delusion group, schizophrenia symptoms were assessed by using the Positive and Negative Syndrome Scale (PANSS) (63) and the Psychotic Symptoms Rating Scale (PSYRATS) (66). PANSS consists of 30 symptoms, rated on a 1 (absent) to 7 (extreme) scale. PANSS P1 indicates the overall delusional severity. PSYRATS consists of the auditory hallucinations subscale and the delusions subscale, with the latter being of interest in this study. The delusions subscale has a score range of 0–24, with items rated on a 0–4 Likert scale. Good psychometrics have been reported for PANSS and PSYRATS, respectively (63, 66, 67).

For the entire sample, the severity of depressive symptoms was measured using the Calgary Depression Scale for Schizophrenia (CDSS) (68, 69). This semi-structured interview scale has been extensively used to assess depression in patients with schizophrenia. It has high inter-rater reliability, sensitivity, specificity, and discriminant and convergent validity (70, 71). The items are rated on a 0–3 scale, and the total score ranges from 0 to 27. Across groups, the level of anxiety was measured by Generalised Anxiety Disorder 7-item scale (GAD-7) (72), which has good validity and reliability (73, 74).

#### Flexibility Measures

All participants completed the following measures assessing three aspects of flexibility.

##### Cognitive Flexibility

The Trail-making task (TMT) was developed to assess an individual's ability to direct thoughts and actions when monitoring alternating tasks (19). In Part A of the TMT, 25 numbered circles (1–25) are mixed and spread about on a white sheet of paper. Participants are asked to connect them in numerical order. In Part B, 13 numbered circles (1–13) and 12 circles with alphabetic letters (A–L) are mixed and spread about on a white sheet of paper of the same size. Participants are asked to connect them alternatively and in ascending order (i.e., 1-A-2-B…). In both parts, participants are asked to complete the task as quickly as possible. An experimenter would time the tasks and point out any respondent's errors immediately. Following the original test manual, cognitive flexibility was calculated as the difference in the completion time for Part B and Part A. A greater TMT difference score indicates poorer cognitive flexibility. TMT is one of the most commonly used measures for cognitive flexibility, and its psychometric properties have been studied widely (75, 76).

##### Explanatory Flexibility

Attributional Style Questionnaire (ASQ) (6, 77) is composed of 12 hypothetical daily events, six positive (e.g., “You get a raise.”) and six negative (e.g., “You go out on a date and it goes badly.”). For each event, participant first provided a perceived cause for its occurrence. Then, they rated the cause on a 1-to-7-Likert scale regarding whether the cause was (1) external vs. internal, (2) temporary vs. stable, (3) context-specific vs. global. ASQ has acceptable-to-good internal consistency and reliability (77). Following Fresco et al. (35, 37, 40), explanatory flexibility was calculated as the standard deviation of the stability and globality items for negative events. A higher score indicates better explanatory flexibility, whereas a lower score indicates inflexibility or rigidity.

##### Belief Flexibility

Belief flexibility was measured by the Maudsley Assessment of Delusions Schedule (MADS) (45) and the Bias Against Disconfirmatory Evidence Task (BADE) (47). Following the MADS interview protocol, a trained experimenter facilitated a discussion about the idiosyncratic affect-laden belief with each participant individually. For the Delusion group, the delusional belief as identified through the PANSS and PSYRATS interview was assessed. For the Depression group and healthy controls, belief flexibility was assessed in the context of explanations of negative daily-life experiences. We first invited the participants to focus on a specific experience that had bothered them personally over the past 2 weeks (for examples, see Table 1). Their interpretation about the event was then elicited. Participants were asked about how strongly they believed in that interpretation; only affect-laden beliefs that were held with more than 50% conviction were further assessed for belief flexibility. The procedure of identification and selection of the idiosyncratic beliefs was comparable across the three groups. As reported by Colbert et al. (78), among non-psychotic individuals, belief flexibility would be more reduced in light of personally meaningful beliefs than standard beliefs (e.g., “The sun will rise tomorrow”). Therefore, we adopted personally meaningful beliefs in this study for better sensitivity. Levels of conviction, preoccupation and distress associated with the belief were rated individually, using the PSYRATS score ranges.

**Table 1:** Examples of idiosyncratic beliefs elicited for the assessment of belief flexibility (MADS).

Delusion group: - People keep coming after me. - People deliberately coughed at me and swore at me. - A device owned by the university is controlling my thoughts.
Depression group: - Peers in my church overlooked me as if I was not there. - My parents did not talk to me, which suggests that I am a disappointment to them. - I failed to take care of my mother well enough.
Control group: - A client who complained about me made trouble out of nothing. - The renovation worker was irresponsible. - My heavy workload has cost my leisure time with friends.

*MADS, the Maudsley Assessment of Delusions Schedule. The sentences are translated from Cantonese to English*.

Once the idiosyncratic belief was identified for the individual participant, the interviewer asked in a semi-structured manner if it was at all possible for the participant to be mistaken about the belief (PM). Then the interviewer proposed a piece of hypothetical and contradictory evidence, which, if true, would convincingly challenge the participant's belief, and assessed their response for the “reaction to hypothetical contradiction” (RTHC) item. Lastly, the interviewer asked the participant to provide an alternative explanation (AE) for their experience (44). The items PM, RTHC, and AE were rated on a dichotomous scale (i.e., flexible/inflexible).

The BADE task is a computerised task that assesses individuals' reappraisal of beliefs in response to disconfirmatory information presented for standardised scenarios. For each scenario, participants were asked to rate the likelihood of four predetermined explanations: one true, one absurd, and two lure explanations. Initially, the scenario appeared to indicate lure explanations. Two new pieces of information about the scenario were then provided one by one. With each new piece of information, the participants could adjust the ratings they had given. Likelihood of explanations was rated on a 0–100 scale. Following Woodward et al. (47), belief flexibility was calculated as the difference score between the third and the first rating of lure explanations, with a higher difference score indicating greater flexibility.

#### Other Measures

General intelligence was estimated using the 4-subtest short form of the Wechsler Adult Intelligence Scale – Third Edition (79). Demographic information was collected via an unpublished questionnaire.

### Procedure

Following informed written consent, participants completed a clinical interview which incorporated the above measures and tasks, as well as brief questions on demographic information. The assessment was conducted in a quiet lab by a graduate-level psychologist or psychiatrist under the supervision of expert interviewers.

### Data Analysis

For group comparisons of categorical variables, we used the chi-square test, followed by *post-hoc* Fisher's exact approach wherever significant differences were identified (80), with alpha level adjusted for Bonferroni correction. Group comparisons of continuous variables were performed on Kruskal-Wallis Test or ANOVA test where appropriate. Kruskal-Wallis comparisons were followed by Dunn's test for *post-hoc* Bonferroni comparisons (81, 82). ANOVA comparisons were followed by Dunnett-Tukey-Kramer test for *post-hoc* comparisons, considering its strength in reducing error rate when sample sizes were unequal yet homogeneous variance was assumed (83). We reported the Pearson correlation between continuous variables and the biserial correlation between categorical variables and continuous variables. Logistic regression and ANCOVA were used to control for potential covariates, including general intelligence and gender. Data analysis was conducted using jamovi (version 1.2) (84) and RStudio (version 1.2.5033) (85), both of which were based on R (version 3.6.3) (86).

## Results

### Sample Characteristics

Demographic characteristics of the three groups are shown in Table 2. There was a significant group difference in gender, with more females in the Depression group than the other groups (*p* < 0.001). There was a significant group difference in estimated intelligence (*p* < 0.001), with the control group outperforming the two clinical groups, and the clinical groups not being different from each other. The three groups were comparable on age and year of education (*p*s > 0.05).

**Table 2:** Demographic characteristics and emotion states.

	**Delusion (*N* = 56)**	**Depression (*N* = 57)**	**Control (*N* = 30)**	**Group comparisons**
Gender (Female/Male)	26/30	47/10	16/14	*χ^2^*_(2,143)_ = 16.9, *p* < 0.001
Age	41.45 (13.83)	45.70 (13.07)	44.87 (14.03)	Kruskal-Wallis χ^2^_(2)_ = 2.88, *p* = 0.237, ε^2^ = 0.02
Education in years	11.66 (3.35)	10.89 (3.61)	11.80 (3.69)	Kruskal-Wallis χ^2^_(2)_ = 2.1, *p* = 0.351, ε^2^ = 0.01
WAIS-III	26.38 (6.38)	28.55 (6.40)	32.69 (6.46)	*F*_(2,137)_ = 8.84, *p* < 0.001
Hospitalisation	1.32 (1.88)	0.42 (0.71)	/	Mann-Whitney *U* = 1,082.50, *p* = 0.001, Cohen's *d* = 0.64
GAD-7	7.65 (5.66)	12.27 (5.28)	1.53 (2.06)	Kruskal-Wallis χ^2^_(2)_ = 59.20, *p* < 0.001, ε^2^ = 0.42
CDSS	3.57 (3.41)	11.14 (5.30)	0.63 (1.13)	Kruskal-Wallis χ^2^_(2)_ = 81.57, *p* < 0.001, ε^2^ = 0.57
**Belief**				
Conviction	3.32 (0.77)	3.30 (0.66)	3.00 (0.53)	Kruskal-Wallis χ^2^_(2)_ = 6.86, *p* = 0.032, ε^2^ = 0.05
Preoccupation	2.26 (1.17)	2.27 (1.04)	1.03 (0.64)	Kruskal-Wallis χ^2^_(2)_ = 29, *p* < 0.001, ε^2^ = 0.17
Distress	2.43 (1.11)	3.26 (0.65)	1.48 (1.15)	Kruskal-Wallis χ^2^_(2)_ = 43.72, *p* < 0.001, ε^2^ = 0.25

*Standard deviations are in parentheses. WAIS-III, Sum of Scaled Scores from the Short Form of Wechsler Adult Intelligence Scale-III; GAD-7, Generalised Anxiety Disorder 7-item Scale; CDSS, Calgary Depression Scale for Schizophrenia*.

Within the Delusion group, the average PANSS scores were as follows: total score = 51.41 (SD = 9.77), Positive subscore = 14.73 (SD = 3.45), Negative subscore = 11.13 (SD = 4.83), General Psychopathology = 26.13 (SD = 6.42), Severity of delusions (P1) = 4.82 (SD = 1.15). The average number of hospitalisations was 1.32 (SD = 1.88). The average antipsychotic dosage (in chlorpromazine equivalents) was 492.04 (SD = 415.64, range 13.03–1,592.84). Within the Depression group, the average BDI-II score was 33.80 (SD = 11.91). The average number of hospitalisations was 0.42 (SD = 0.71), which was significantly lower than the Delusion group (Mann-Whitney *U* = 1,082.50, *p* = 0.001, Cohen's *d* = 0.64). The average antidepressant dosage (in Fluoxetine equivalents) was 37.76 (SD = 26.13, range 4.98–97.06).

As shown in Table 2, there were significant overall group differences on GAD-7 and CDSS (*ps* < 0.001). On both measures, the Depression group was higher than the Delusion group (*ps* < 0.001), which in turn was higher than healthy controls (*ps* ≤ 0.001).

### Group Comparisons of Cognitive Flexibility

Means and SDs of flexibility indices are shown in Table 3. There was a significant main effect of group. Pairwise comparisons revealed that cognitive flexibility was significantly higher in controls than the two clinical groups (mean difference with the Delusion group = 2.67, *p* = 0.011; mean difference with the Depression group = 3.04, *p* = 0.004); whereas the two clinical groups did not differ significantly (*p* = 0.981). In follow-up analyses, the main effect of group disappeared when controlling for estimated IQ [*F*_(2,131)_ = 1.80, *p* = 0.169, partial η^2^ = 0.027].

**Table 3:** Means and SDs of flexibility indices.

	**Delusion**			**Depression**			**Control**			**Group Comparisons**	***post-hoc* pairwise comparisons**
	***N***	**M**	**SD**	***N***	**M**	**SD**	***N***	**M**	**SD**		
**Cognitive flexibility**											
TMT B-A	54	49.64	34.59	52	48.50	29.29	30	32.30	18.86	Kruskal-Wallis χ^2^_(2)_ = 10.13, *p* = 0.006, ε^2^ = 0.08	Delusion less flexible than Control, Depression less flexible than Control
**Explanatory flexibility**											
ASQ	55	1.45	0.50	56	1.45	0.63	29	1.44	0.54	*F*_(2,137)_ = 0.01, *p* = 0.995	
**Belief flexibility**											
**PM**										χ^2^_(2,142)_ = 5.31, *p* = 0.070	
Inflexible	32			29			9				
Flexible	24			28			20				
**RTHC**										χ^2^_(2,141)_ = 17.14, *p* < 0.001	Delusion less flexible than Depression and Control
Inflexible	46			33			11				
Flexible	10			23			18				
**AE**										χ^2^_(2,141)_ = 8.76, *p* = 0.013	Delusion less flexible than Control
Inflexible	36			27			9				
Flexible	20			29			20				
BADE Dif	45	23.19	23.62	55	16.39	16.95	30	22.32	17.51	Kruskal-Wallis χ^2^_(2)_ = 2.73, *p* = 0.255, ε^2^ = 0.02	

*TMT, Trail-Making Test; ASQ, standard deviation for negative events on the Attributional Style Questionnaire; PM, possibility of being mistaken; RTHC, reaction to hypothetical contradiction; AE, alternative explanation; BADE, Bias Against Disconfirmatory Evidence Task; Dif, difference score; EI, evidence integration; CV, conservatism. Pairwise comparions were Bonferroni corrected*.

### Group Comparisons of Explanatory Flexibility

There was no significant difference in explanatory flexibility across groups (Table 3).

### Group Comparisons of Belief Flexibility

Dimensions of beliefs across groups are shown in Table 2. On MADS, there was a significant main effect of group in RTHC and AE, but not PM (Table 3). The Delusion group was significantly less flexible on the RTHC item compared to the other groups [Fisher's exact test = 3.17, odds ratio = 3.21 (95% CI = 1.35–7.63), *p* = 0.012 with Depression and Fisher's exact test = 7.31, odds ratio = 7.53 (95% CI = 2.73–20.8), *p* < 0.001 with Controls]. There was no significant difference between the Depression and Control groups (*p* = 0.073). The ability to generate alternative explanations was significantly reduced in the Delusion group compared to the Control group [Fisher's exact test = 3.93, odds ratio = 4 (95% CI = 1.53–10.4), *p* = 0.006], whereas no significant difference was found between the two clinical groups (*p* = 0.127) or between the Depression and Control groups (*p* = 0.167).

As there were significant group differences in gender, estimated IQ and emotional states, we analysed the significant associations again controlling for these variables. The results on belief flexibility remained significant (*ps* < 0.05).

There was no group difference on the BADE task performance.

### Association Between Flexibility Indices

Associations between flexibility indices are shown in Table 4. In the entire sample (*N* = 143), the completion time difference on TMT was negatively correlated with BADE difference score and AE, indicating that higher cognitive flexibility was associated with higher belief flexibility (as measured by BADE and MADS).

**Table 4:** Correlations between flexibility indices within each group.

	**All (*****N*** **=** **143)**		**Delusion (*****N*** **=** **56)**		**Depression (*****N*** **=** **57)**		**Control (*****N*** **=** **30)**	
	**TMT**	**ASQ**	**TMT**	**ASQ**	**TMT**	**ASQ**	**TMT**	**ASQ**
ASQ	0	/	0.22	/	−0.13	/	−0.23	/
PM	−0.14	−0.21	−0.13	−0.46^**^	−0.01	−0.14	−0.17	0.06
RTHC	−0.18	−0.13	−0.12	−0.04	0.03	−0.19	−0.50^*^	−0.22
AE	−0.30^**^	−0.19	−0.24	−0.37^*^	−0.30	−0.12	−0.24	−0.10
BADE Dif	−0.21^*^	0.08	−0.24	−0.07	−0.11	0.10	−0.39^*^	0.37^*^

Pearson correlations (without Bonferroni correction) were reported among BADE, ASQ, and TMT, whereas biserial correlation among PM, RTHC, AE, ASQ, and TMT. TMT, Trail-Making Test difference score; ASQ, standard deviation for negative events on the Attributional Style Questionnaire; PM, possibility of being mistaken; RTHC, reaction to hypothetical contradiction; AE, alternative explanation; BADE, Bias Against Disconfirmatory Evidence Task; Dif, difference score; EI, evidence integration; CV, conservatism;

* *, p < 0.05*.

** *, p < 0.01*.

Among healthy controls (*N* = 30), TMT difference score was negatively correlated with BADE performance and RTHC, which indicated that higher cognitive flexibility was associated with higher belief flexibility (as measured by BADE and MADS). However, similar associations were not found in the two clinical groups. Across groups, explanatory flexibility was not correlated with cognitive flexibility (*ps* > 0.05). While explanatory flexibility was positively correlated with belief flexibility (on BADE only) in the Control group (*p* < 0.05), it was negatively correlated with belief flexibility (on MADS only) in the Delusion group (*ps* < 0.05). All correlation coefficients between flexibility indices in the Depression group were small-to-moderate and non-significant.

## Discussion

The current study compared flexibility indices in patients with delusions, patients with depression, and healthy controls. We found that:

1. Compared to healthy controls, cognitive flexibility was reduced in both the Delusion group and the Depression group.
2. The three groups did not differ in explanatory flexibility.
3. Compared to controls and the Depression group, belief flexibility as measured by interview items was reduced in the Delusion group, but not belief flexibility as measured by the BADE task.

Our finding that both the Delusion group and the Depression group had reduced cognitive flexibility was consistent with previous studies (31), and lends support to the argument that cognitive flexibility may be generally associated with psychopathologies regardless of the diagnostic label (22–24). It is of note that the difference between clinical groups and healthy controls was no longer significant after controlling for estimated IQ. Executive functions such as updating and set-shifting that are crucial for TMT performance were shown to be also associated with fluid and crystallised intelligence (87, 88), suggesting a considerable shared variance between the two. Since general intelligence is typically lower in schizophrenia and considered a risk factor for disease (89–91), the extent to which such shared variance also overlaps with a genuine effect of the psychopathology remains speculative.

Our hypothesis about explanatory flexibility was partially supported. The comparable levels of explanatory flexibility between the Delusion group and the Depression group replicated Silverman and Peterson (38). As patients with psychotic depression were excluded from this study, and the Delusion group had a low CDSS score, the compromised explanatory flexibility in the Delusion group cannot be explained by depressive symptoms. Therefore, our results added to the accumulating evidence that explanatory flexibility is not unique to MDD but can be seen in other disorders as well. However, while the level of explanatory flexibility in our Depression group fell within the range of other MDD samples (39, 40), our healthy controls manifested a comparable level of explanatory flexibility, too. This initial finding, which potentially suggests non-specificity of explanatory flexibility, warrants further testing using a larger sample.

With regard to belief flexibility as measured by the clinical interview, we compared the responses based on delusions in the Delusion group, negative thinking in the Depression group, and a negative and personally significant belief for the healthy controls. Results on PM and RTHC were different. This was consistent with previous research (46, 92), suggesting that RTHC might rely on a different, if not deeper, level of reflections than PM. Even on such a stringent test using idiosyncratic beliefs that are salient to the individual participants, the Delusion group still manifested the lowest belief flexibility on two of the three MADS variables. While inflexible thinking has been studied in depression literature [e.g., (14, 37, 93)], to our knowledge, this was the first study that directly compared individuals with psychotic delusions with individuals with MDD. Our finding lends support to the specificity of belief flexibility (measured on MADS) to patients with delusions. Our finding was consistent with our recent treatment trial (62) where change in belief flexibility did not moderate improvement in depression. However, the BADE difference score appeared to be smaller in the Depression group than the other two groups (albeit not statistically significant), which raises the possibility that the Depression group may be less flexible on the BADE task. Together with Everaert et al. (55), which found a similar pattern in a community sample, the possibility that individuals with depression may have reduced belief flexibility (although not as low as individuals with delusions) cannot be completely ruled out and is worth further investigation.

We explored the associations between flexibility constructs, which should be interpreted with caution, given the small group sizes. We found that higher cognitive flexibility was associated with higher belief flexibility, especially in the control group. Such association was consistent with Eifler et al. (59) and Riccaboni et al. (60). Since the association was evident for both interview and standardised task measures of belief flexibility, it is not likely to be an artefact of the nature of the task. As argued by Baddeley (94) and Banich (95), effortful modulation of mental processes (as opposed to autonomous, routine ones) may require activation of executive functioning. Such correlation was weaker (and not significant) in the clinical groups, which could possibly be attributed to the poorer cognitive flexibility in these groups. The hypothesis that cognitive flexibility might underlie other forms of flexibility was not fully supported, as cognitive flexibility was not associated with explanatory flexibility across groups. The association between explanatory flexibility and belief flexibility was equivocal, with a positive association manifested in the Control group, and a negative association in the Delusion group. What explanatory flexibility entails remains unclear. On the one hand, there is preliminary evidence that explanatory flexibility decreases when the negative mood is induced, leading to the argument that explanatory flexibility may be a result of a negative mood state (34, 40). On the other hand, the way explanatory flexibility is measured (i.e., standard deviation of ASQ item responses) may reflect participants' extreme responding and jumping-to-conclusions tendencies, which are particularly marked among patients with delusions and have been shown to be associated with lack of belief flexibility (12, 13, 42). Further research on explanatory flexibility will enhance our understanding of this construct and its role in making (re-)appraisals across psychiatric groups.

There are several limitations to the study. Firstly, the sample sizes were unequal, which might compromise the statistical power and lead to a type II error (96). We chose tests less impacted by unequal group sizes, but the results await replication. Secondly, this was a cross-sectional study, leading to no causality findings of the relationship between flexibility and diagnosis or symptoms. Longitudinal research would further shed light on whether flexibility, or in this case the lack thereof, leads to, perpetuates, results from, or only co-occurs with any single symptom or a particular diagnosis. Thirdly, we included the most commonly used measures of flexibility, which led to an imbalance in the number of measures across flexibility constructs. In particular, even though the TMT task is widely used to measure flexibility, this task is dependent upon motor speed function, which is affected in these disorders. It is unsure whether including another measure of cognitive flexibility, such as the Wisconsin card sorting task, would further strengthen the investigation. Our results cannot be generalisable to other measures of flexibility. Lastly, since we only included patients with schizophrenia-spectrum disorders and patients with major depressive disorder, how these flexibility constructs compare across other psychiatric groups remain to be tested.

This was the first empirical study investigating cognitive flexibility, explanatory flexibility, and belief flexibility across psychiatric groups, in comparison with a non-clinical group. We found an effect of clinical status on the more fundamental level of flexibility, which might be confounded by the group difference in estimated intelligence. Reduced belief flexibility tended to be shown in patients with delusions only, calling for more research on its specificity.

## Data Availability Statement

The raw data supporting the conclusions of this article will be made available by the authors, without undue reservation.

## Ethics Statement

The studies involving human participants were reviewed and approved by the Joint Chinese University of Hong Kong - New Territories East Cluster Clinical Research Ethics Committee and the New Territories West Cluster Research Ethics Committee. The patients/participants provided their written informed consent to participate in this study.

## Author Contributions

CZ and SS contributed to the study design. CZ came up with the first draft of the manuscript, which was reviewed and approved by all authors. All authors contributed to data collection and analysis.

## Conflict of Interest

The authors declare that the research was conducted in the absence of any commercial or financial relationships that could be construed as a potential conflict of interest.

## References

[1] Beck AT. The past and future of cognitive therapy. J Psychother Pract Res. (1997) 6:276–84. 9292441 PMC3330473

[2] Butler AC Chapman JE Forman EM Beck AT. The empirical status of cognitive-behavioral therapy: a review of meta-analyses. Clin Psychol Rev. (2006) 26:17–31. 10.1016/j.cpr.2005.07.003 16199119

[3] Ellis A The revised ABC's of rational-emotive therapy (RET). J Ration Emot Cogn Behav Ther. (1991) 9:139–72. 10.1007/BF01061227

[4] Hollon SD Beck AT Cognitive and cognitive-behavioral therapies. In: Bergin AE Garfield SL editors. Handbook of Psychotherapy and Behavior Change. New York, NY: John Wiley & Sons (2013). p. 393–442.

[5] Wells A Metacognitive Therapy for Anxiety and Depression. New York, NY: Guilford Press (2011). 316 p.

[6] Seligman ME Abramson LY Semmel A von Baeyer C. Depressive attributional style. J Abnorm Psychol. (1979) 88:242–7. 10.1037/0021-843X.88.3.242 500951

[7] Cartwright-Hatton S Wells A. Beliefs about worry and intrusions: the meta-cognitions questionnaire and its correlates. J Anxiety Disord. (1997) 11:279–96. 10.1016/S0887-6185(97)00011-X 9220301

[8] Wells A Meta-cognition and worry: a cognitive model of generalised anxiety disorder. Behav Cogn Psychother. (1995) 23:301–20. 10.1017/S1352465800015897

[9] Beck AT Davis DD Freeman A editors. Cognitive Therapy of Personality Disorders. New York: Guilford Publications (2015). 506 p.

[10] Egan SJ Piek JP Dyck MJ Rees CS. The role of dichotomous thinking and rigidity in perfectionism. Behav Res Ther. (2007) 45:1813–22. 10.1016/j.brat.2007.02.002 17382290

[11] Garety PA Hemsley DR Wessely S. Reasoning in deluded schizophrenic and paranoid patients: biases in performance on a probabilistic inference task. J Nerv Ment Dis. (1991) 179:194–201. 10.1097/00005053-199104000-00003 2007889

[12] So SH Siu NY Wong HL Chan W Garety PA. 'Jumping to conclusions' data-gathering bias in psychosis and other psychiatric disorders -two meta-analyses of comparisons between patients and healthy individuals. Clin Psychol Rev. (2016) 46:151–67. 10.1016/j.cpr.2016.05.001 27216559

[13] McLean BF Mattiske JK Balzan RP. Association of the jumping to conclusions and evidence integration biases with delusions in psychosis: a detailed meta-analysis. Schizophr Bull. (2017) 43:344–54. 10.1093/schbul/sbw056 27169465 PMC5605251

[14] Stange JP Alloy LB Fresco DM. Inflexibility as a vulnerability to depression: a systematic qualitative review. Clin Psychol Sci Pract. (2017) 24:245–76. 10.1111/cpsp.12201 29038622 PMC5640320

[15] Dajani DR Uddin LQ. Demystifying cognitive flexibility: implications for clinical and developmental neuroscience. Trends Cogn Sci. (2015) 38:571–8. 10.1016/j.tins.2015.07.003 26343956 PMC5414037

[16] Armbruster DJ Ueltzhöffer K Basten U Fiebach CJ. Prefrontal cortical mechanisms underlying individual differences in cognitive flexibility and stability. J Cogn Neurosci. (2012) 24:2385–99. 22905818 10.1162/jocn_a_00286

[17] Scott WA Cognitive complexity and cognitive flexibility. Sociometry. (1962) 1:405–14. 10.2307/2785779

[18] Merriam EP Thase ME Haas GL Keshavan MS Sweeney JA. Prefrontal cortical dysfunction in depression determined by Wisconsin card sorting test performance. Am J Psychiatry. (1999) 156:780–2. 10327916 10.1176/ajp.156.5.780

[19] Armitage SG An analysis of certain psychological tests used for the evaluation of brain injury. Psychol Monogr. (1946) 60:30–34.

[20] Owen AM Roberts AC Polkey CE Sahakian BJ Robbins T.W. Extra-dimensional versus intra-dimensional set shifting performance following frontal lobe excisions, temporal lobe excisions or amygdalo-hippocampectomy in man. Neuropsychologia. (1991) 29:993–1006. 10.1016/0028-3932(91)90063-E 1762678

[21] Robbins TW James M Owen AM Sahakian BJ McInnes L Rabbitt P. Cambridge neuropsychological test automated battery (CANTAB): a factor analytic study of a large sample of normal elderly volunteers. Dement Geriatr Cogn Disord. (1994) 5:266–81. 10.1159/000106735 7951684

[22] Bloemen AJ Oldehinkel AJ Laceulle OM Ormel J Rommelse NN Hartman CA The association between executive functioning and psychopathology: general or specific? Psychol Med. (2018) 48:1787–94. 10.1017/S0033291717003269 29521611

[23] Morris L Mansell W A systematic review of the relationship between rigidity/flexibility and transdiagnostic cognitive and behavioral processes that maintain psychopathology. J Exp Psychopathol. (2018) 9:1–40. 10.1177/2043808718779431

[24] Snyder HR Miyake A Hankin BL. Advancing understanding of executive function impairments and psychopathology: bridging the gap between clinical and cognitive approaches. Front Psychol. (2015) 6:328. 10.3389/fpsyg.2015.00328 25859234 PMC4374537

[25] Koren D Seidman LJ Harrison RH Lyons MJ Kremem WS Caplan B. Factor structure of the Wisconsin card sorting test: dimensions of deficit in schizophrenia. Neuropsychology. (1998) 12:289–302. 10.1037/0894-4105.12.2.289 9556775

[26] Laere E Tee SF Tang PY. Assessment of cognition in schizophrenia using trail making test: a meta-analysis. Psychiatry Investig. (2018) 15:945–55. 10.30773/pi.2018.07.22 30223641 PMC6212701

[27] Geurts HM Corbett B Solomon M. The paradox of cognitive flexibility in autism. Trends Cogn Sci. (2009) 13:74–82. 10.1016/j.tics.2008.11.006 19138551 PMC5538880

[28] Sanders J Johnson KA Garavan H Gill M Gallagher L A review of neuropsychological and neuroimaging research in autistic spectrum disorders: attention, inhibition and cognitive flexibility. Res Autism Spectr Disord. (2008) 2:1–16. 10.1016/j.rasd.2007.03.005

[29] Gruner P Pittenger C. Cognitive inflexibility in obsessive-compulsive disorder. Neuroscience. (2017) 345:243–55. 10.1016/j.neuroscience.2016.07.030 27491478 PMC5288350

[30] Mahurin RK Velligan DI Hazleton B Davis JM Eckert S Miller AL. Trail making test errors and executive function in schizophrenia and depression. Clin Neuropsychol. (2006) 20:271–88. 10.1080/13854040590947498 16690547

[31] Moritz S Birkner C Kloss M Jahn H Hand I Haasen C. Executive functioning in obsessive-compulsive disorder, unipolar depression, and schizophrenia. Arch Clin Neuropsychol. (2002) 17:477–83. 10.1093/arclin/17.5.477 14592001

[32] Best JR Miller PH. A developmental perspective on executive function. Child Dev. (2010) 81:1641–60. 10.1111/j.1467-8624.2010.01499.x 21077853 PMC3058827

[33] Garon N Bryson SE Smith IM. Executive function in preschoolers: a review using an integrative framework. Psychol Bull. (2008) 134:31–60. 10.1037/0033-2909.134.1.31 18193994

[34] Fresco DM Heimberg RG Abramowitz A Bertram TL. The effect pf a negative mood priming challenge on dysfunctional attitudes, explanatory style, and explanatory flexibility. Br J Clin Psychol. (2006) 45:167–83. 10.1348/014466505X35137 16719978

[35] Fresco DM Rytwinski NK Craighead LW Explanatory flexibility and negative life events interact to predict depression symptoms. J Soc Clin Psychol. (2007) 26:595–608. 10.1521/jscp.2007.26.5.595

[36] Joseph JS Gray MJ The utility of measuring explanatory flexibility in PTSD research. Cognit Ther Res. (2011) 35:372–80. 10.1007/s10608-010-9301-7

[37] Fresco DM Williams NL Nugent NR Flexibility and negative affect: examining the associations of explanatory flexibility and coping flexibility to each other and to depression and anxiety. Cognit Ther Res. (2006) 30:201–10. 10.1007/s10608-006-9019-8

[38] Silverman RJ Peterson C Explanatory style of schizophrenic and depressed outpatients. Cognit Ther Res. (1993) 17:457–70. 10.1007/BF01173057

[39] Moore MT Fresco DM Schumm JA Dobson KS. Change in explanatory flexibility and explanatory style in cognitive therapy and its components. Cognit Ther Res. (2017) 41:519–29. 10.1007/s10608-016-9825-626696692

[40] Fresco DM Mennin DS Moore MT Heimberg RG Hambrick J Changes in explanatory flexibility among individuals with generalized anxiety disorder in an emotion evocation challenge. Cognit Ther Res. (2014) 38:416–27. 10.1007/s10608-014-9601-4

[41] Lackner RJ Moore MT Minerovic JR Fresco DM. Explanatory flexibility and explanatory style in treatment-seeking patients with axis I psychopathology. Cognit Ther Res. (2015) 39:736–43. 10.1007/s10608-015-9702-8 26696692 PMC4684804

[42] Garety PA Freeman D Jolley S Dunn G Bebbington PE Fowler DG. Reasoning, emotions, and delusional conviction in psychosis. J Abnorm Psychol. (2005) 114:373–84. 10.1037/0021-843X.114.3.373 16117574

[43] Garety PA Ward T Rus-Calafell M Beyond belief-new approaches to the treatment of paranoia. In: Badcock JC, Paulik G. A Clinical Introduction to Psychosis. London: Academic Press (2020). p. 591–613.

[44] Freeman D Garety PA Fowler D Kuipers E Bebbington PE Dunn G. Why do people with delusions fail to choose more realistic explanations for their experiences? An empirical investigation. J Consult Clin Psychol. (2004) 72:671–80. 10.1037/0022-006X.72.4.671 15301652

[45] Wessely S Buchanan A Reed A Cutting J Everitt B Garety P. Acting on delusions. I: Prevalence. Br J Psychiatry. (1993) 163:69–76. 10.1192/bjp.163.1.69 8353703

[46] So SH Freeman D Dunn G Kapur S Kuipers E Bebbington P. Jumping to conclusions, a lack of belief flexibility and delusional conviction in psychosis: a longitudinal investigation of the structure, frequency, and relatedness of reasoning biases. J Abnorm Psychol. (2012) 121:129–39. 10.1037/a0025297 21910515 PMC3283358

[47] Woodward TS Moritz S Menon M Klinge R. Belief inflexibility in schizophrenia. Cogn Neuropsychiatry. (2008) 13:267–77. 10.1080/13546800802099033 18484291

[48] Speechley WJ Ngan ET Moritz S Woodward TS Impaired evidence integration and delusions in schizophrenia. J Exp Psychopathol. (2012) 3:688–701. 10.5127/jep.018411

[49] van der Gaag M Schütz C ten Napel A Landa Y Delespaul P Bak M. Development of the Davos assessment of cognitive biases scale (DACOBS). Schizophr Res. (2013) 144:63–71. 10.1016/j.schres.2012.12.010 23332365

[50] Beck AT Baruch E Balter JM Steer RA Warman DM. A new instrument for measuring insight: the Beck cognitive insight scale. Schizophr Res. (2004) 68:319–29. 10.1016/S0920-9964(03)00189-0 15099613

[51] Hardy A Tolmeijer E Edwards V Ward T Freeman D Emsley R Measuring reasoning in paranoia: development of the fast and slow thinking questionnaire. Schizophr Bull Open. (2020) 1:sgaa035 10.1093/schizbullopen/sgaa035

[52] Zhu C Sun X So SH. Associations between belief inflexibility and dimensions of delusions: a meta-analytic review of two approaches to assessing belief flexibility. Br J Clin Psychol. (2018) 57:59–81. 10.1111/bjc.12154 28805246

[53] Penzenstadler L Chatton A Huguelet P Lecardeur L Bartolomei J Brazo P. Does change over time in delusional beliefs as measured with PDI predict change over time in belief flexibility measured with MADS? Psychiatr Q. (2019) 90:693–702. 10.1007/s11126-019-09659-8 31338790

[54] Sanford N Veckenstedt R Moritz S Balzan RP Woodward TS. Impaired integration of disambiguating evidence in delusional schizophrenia patients. Psychol Med. (2014) 44:2729–38. 10.1017/S0033291714000397 25065271

[55] Everaert J Bronstein MV Cannon TD Joormann J Looking through tinted glasses: depression and social anxiety are related to both interpretation biases and inflexible negative interpretations. Clin Psychol Sci. (2018) 6:517–28. 10.1177/2167702617747968

[56] Eisenacher S Zink M Holding on to false beliefs: the bias against disconfirmatory evidence over the course of psychosis. J Behav Ther Exp Psychiatry. (2017) 56:79–89. 10.1016/j.jbtep.2016.08.015 27608522

[57] Cuthbert BN Insel TR. Toward the future of psychiatric diagnosis: the seven pillars of RDoC. BMC Med. (2013) 11:126. 10.1186/1741-7015-11-126 23672542 PMC3653747

[58] Insel TR Cuthbert BN Garvey MA Heinssen RK Pine DS Quinn KJ Research domain criteria (RDoC): toward a new classification framework for research on mental disorders. Am J Psychiatry. (2010) 167:748–51. 10.1176/appi.ajp.2010.09091379 20595427

[59] Eifler S Rausch F Schirmbeck F Veckenstedt R Englisch S Meyer-Lindenberg A. Neurocognitive capabilities modulate the integration of evidence in schizophrenia. Psychiatry Res. (2014) 219:72–8. 10.1016/j.psychres.2014.04.056 24880580

[60] Riccaboni R Fresi F Bosia M Buonocore M Leiba N Smeraldi E. Patterns of evidence integration in schizophrenia and delusion. Psychiatry Res. (2012) 200:108–14. 10.1016/j.psychres.2012.04.005 22578403

[61] Moritz S Veckenstedt R Hottenrott B Woodward TS Randjbar S Lincoln TM. Different sides of the same coin? Intercorrelations of cognitive biases in schizophrenia. Cogn Neuropsychiatry. (2010) 15:406–21. 10.1080/13546800903399993 20146127

[62] So SH Chan GH Wong CK Ching EW Lee SS Wong BC. A randomised controlled trial of metacognitive training for psychosis, depression, and belief flexibility. J Affect Disord. (2021) 279:388–97. 10.1016/j.jad.2020.09.126 33099054

[63] Kay SR Fiszbein A Opler LA. The positive and negative syndrome scale (PANSS) for schizophrenia. Schizophr Bull. (1987) 13:261–76. 10.1093/schbul/13.2.261 3616518

[64] Beck AT Steer RA Brown G Manual for the Beck Depression Inventory-II. San Antonio, TX: Psychological Corporation (1996).

[65] So E Kam I Leung CM Chung D Liu Z Fong S The Chinese-bilingual SCID-I/P project: stage 1–reliability for mood disorders and schizophrenia. Hong Kong J Psychiatry. (2003) 13:7–19.

[66] Haddock G McCarron J Tarrier N Faragher EB. Scales to measure dimensions of hallucinations and delusions: the psychotic symptom rating scales (PSYRATS). Psychol Med. (1999) 29:879–89. 10.1017/S0033291799008661 10473315

[67] Drake R Haddock G Tarrier N Bentall R Lewis S. The psychotic symptom rating scales (PSYRATS): their usefulness and properties in first episode psychosis. Schizophr Res. (2007) 89:119–22. 10.1016/j.schres.2006.04.024 17095193

[68] Addington D Addington J Schissel B. A depression rating scale for schizophrenics. Schizophr Res. (1990) 3:247–51. 10.1016/0920-9964(90)90005-R 2278986

[69] Addington D Addington J Maticka-Tyndale E Joyce J. Reliability and validity of a depression rating scale for schizophrenics. Schizophr Res. (1992) 6:201–8. 10.1016/0920-9964(92)90003-N 1571313

[70] Addington D Addington J Maticka-Tyndale E Assessing depression in schizophrenia: the calgary depression scale. Br J Psychiatry. (1993) 163:39–44. 10.1192/S0007125000292581 8110442

[71] Addington D Addington J Maticka-Tyndale E. Specificity of the calgary depression scale for schizophrenics. Schizophr Res. (1994) 11:239–44. 10.1016/0920-9964(94)90017-5 8193062

[72] Spitzer RL Kroenke K Williams JB Löwe B. A brief measure for assessing generalized anxiety disorder: the GAD-7. Arch Intern Med. (2006) 166:1092–7. 10.1001/archinte.166.10.1092 16717171

[73] Jordan P Shedden-Mora MC Löwe B. Psychometric analysis of the generalized anxiety disorder scale (GAD-7) in primary care using modern item response theory. PLoS ONE. (2017) 12:e0182162. 10.1371/journal.pone.0182162 28771530 PMC5542568

[74] Rutter LA Brown TA. Psychometric properties of the generalised anxiety disorder scale-7 (GAD-7) in outpatients with anxiety and mood disorders. J Psychopathol Behav Assess. (2017) 39:140–6. 10.1007/s10862-016-9571-9 28260835 PMC5333929

[75] Armitage SG An analysis of certain psychological tests used for the evaluation of brain injury. Psychol Monogr. (1946) 60:i-48. 10.1037/h0093567

[76] Tombaugh TN. Trail making test A and B: normative data stratified by age and education. Arch Clin Neuropsychol. (2004) 19:203–14. 10.1016/S0887-6177(03)00039-8 15010086

[77] Peterson C Semmel A Von Baeyer C Abramson LY Metalsky GI Seligman ME The attributional style questionnaire. Cognit Ther Res. (1982) 6:287–99. 10.1007/BF01173577

[78] Colbert SM Peters ER Garety PA. Delusions and belief flexibility in psychosis. Psychol Psychother. (2010) 83:45–57. 10.1348/147608309X467320 19712542

[79] Wechsler D Wechsler Adult Intelligence scale-Third Edition (WAIS-III). San Antonio, TX: NCS Pearson (1997).

[80] Shan G Gerstenberger S. Fisher's exact approach for post hoc analysis of a chi-squared test. PLoS ONE. (2017) 12:e0188709. 10.1371/journal.pone.0188709 29261690 PMC5737889

[81] Dunn OJ Multiple comparisons using rank sums. Technometrics. (1964) 6:241–52. 10.1080/00401706.1964.10490181

[82] Howell DC Statistical Methods for Psychology. Cengage Learning. Belmont, CA: Wadsworth (2011).

[83] Dunnett CW Pairwise multiple comparisons in the unequal variance case. J Am Stat Assoc. (1980) 75:789–95. 10.1080/01621459.1980.10477551

[84] The jamovi project jamovi. (Version 1.2) [Computer Software]. (2020). Available online at: https://www.jamovi.org (accessed June 11, 2020).

[85] R Studio Team RStudio: Integrated Development for R. Boston, MA: R Studio Inc (2019) Available online at: http://www.rstudio.com/ (accessed June 11, 2020).

[86] R Core Team R: A Language and Environment for Statistical Computing. Vienna: R Core Team (2020). Available online at: https://www.R-project.org/

[87] Colzato LS Van Wouwe NC Lavender TJ Hommel B. Intelligence and cognitive flexibility: fluid intelligence correlates with feature “unbinding” across perception and action. Psychon Bull Rev. (2006) 13:1043–8. 10.3758/BF03213923 17484433

[88] Friedman NP Miyake A Corley RP Young SE DeFries JC Hewitt JK. Not all executive functions are related to intelligence. Psychol Sci. (2006) 17:172–9. 10.1111/j.1467-9280.2006.01681.x 16466426

[89] Hasselbalch BJ Knorr U Kessing LV. Cognitive impairment in the remitted state of unipolar depressive disorder: a systematic review. J Affect Disord. (2011) 134:20–31. 10.1016/j.jad.2010.11.011 21163534

[90] Kahn RS Keefe RS. Schizophrenia is a cognitive illness: time for a change in focus. JAMA Psychiatry. (2013) 70:1107–12. 10.1001/jamapsychiatry.2013.155 23925787

[91] MacKenzie LE Uher R Pavlova B. Cognitive performance in first-degree relatives of individuals with vs without major depressive disorder: a meta-analysis. JAMA Psychiatry. (2019) 76:297–305. 10.1001/jamapsychiatry.2018.3672 30586133 PMC6439825

[92] Garety PA Gittins M Jolley S Bebbington P Dunn G Kuipers E. Differences in cognitive and emotional processes between persecutory and grandiose delusions. Schizophr Bull. (2013) 39:629–39. 10.1093/schbul/sbs059 22499781 PMC3627767

[93] Davis RN Nolen-Hoeksema S Cognitive inflexibility among ruminators and nonruminators. Cognit Ther Res. (2000) 24:699–711. 10.1023/A:1005591412406

[94] Baddeley A. The central executive: a concept and some misconceptions. J Int Neuropsychol Soc. (1998) 4:523–6. 10.1017/S135561779800513X 9745242

[95] Banich MT Executive function: the search for an integrated account. Curr Dir Psychol Sci. (2009) 18:89–94. 10.1111/j.1467-8721.2009.01615.x

[96] Rusticus SA Lovato CY Impact of sample size and variability on the power and type I error rates of equivalence tests: a simulation study. Pract Assess Res Eval. (2014) 19:11 10.7275/4s9m-4e81

